# New field wind manipulation methodology reveals adaptive responses of steppe plants to increased and reduced wind speed

**DOI:** 10.1186/s13007-020-00705-2

**Published:** 2021-01-06

**Authors:** Shudong Zhang, Guofang Liu, Qingguo Cui, Zhenying Huang, Xuehua Ye, Johannes H. C. Cornelissen

**Affiliations:** 1 State Key Laboratory of Vegetation and Environmental Change, Institute of Botany, Chinese Academy of Sciences, Beijing, 100093 People’s Republic of China; 2 University of Chinese Academy of Sciences, Beijing, 100049 People’s Republic of China; 3 Systems Ecology, Department of Ecological Science, Vrije Universiteit, De Boelelaan 1085, 1081 HV Amsterdam, The Netherlands

**Keywords:** Biomass and allocations, Ecological method, Mechanical properties, Mu Us Sandland, Plant size, Wind funneling

## Abstract

**Background:**

Wind strongly impacts plant growth, leaf traits, biomass allocation, and stem mechanical properties. However, whether there are common whole-plant wind responses among different plant species is still unclear. We tested this null hypothesis by exposing four eudicot steppe species to three different wind treatments in a field experiment: reduced wind velocity using windbreaks, ambient wind velocity, and enhanced wind velocity through a novel methodology using wind-funneling baffles.

**Results:**

Across the four species, wind generally decreased plant height, projected crown area, and stepwise bifurcation ratio, and increased root length and stem base diameter. In contrast, the response patterns of shoot traits, especially mechanical properties, to wind velocity were idiosyncratic among species. There was no significant difference in total biomass among different treatments; this might be because the negative effects on heat dissipation and photosynthesis of low wind speed during hot periods, could counteract positive effects during favorable cooler periods.

**Conclusions:**

There are common wind response patterns in plant-size-related traits across different steppe species, while the response patterns in shoot traits vary among species. This indicates the species-specific ways by which plants balance growth and mechanical support facing wind stress. Our new field wind manipulation methodology was effective in altering wind speed with the intended magnitude. Especially, our field wind-funneling baffle system showed a great potential for use in future field wind velocity enhancement. Further experiments are needed to reveal how negative and positive effects play out on whole-plant performance in response to different wind regimes, which is important as ongoing global climatic changes involve big changes in wind regimes.

## Background

Almost all terrestrial plants face wind stress, especially in mountain and coastal ecosystems, plateaus and other inland ecosystems where wind has sufficient open space or natural funneling to gain in force [1–4]. Wind is a major source of mechanical loading and leaf temperature regime on plants, by which it has a major impact on plant growth, morphology, physiology, dispersal and ecology [3, 5–7]. Almost all plants have to balance five major requirements throughout their lifetime: photosynthesis, water transport, growth, reproduction, and mechanical support [8]. Wind may affect all these five requirements. First, in the natural environment, wind is the main source of mechanical perturbation. The mechanical signals that plants perceive imposed by wind can induce thigmomorphogenesis, which may alter plant growth patterns and cause lower stature, thicker stem, and smaller shoots [5, 9–13]. Additionally, mechanical stress can reduce plant growth [14, 15], increase the root/shoot ratio [13, 16], and change leaf properties including a reduction in the number of leaves, individual leaf area or dry mass and petiole length, and increase leaf thickness and flexibility [5, 7, 17–19]. Second, besides creating stressful drag force, wind also influences photosynthesis and transpiration of plants in different ways depending on plant traits, wind intensities and ambient temperatures. Photosynthetic rates will decrease at (very) low wind speed due to the increase in the leaf boundary layer and the consequent reduction in the diffusive resistance for carbon dioxide; at high ambient air temperatures a thick boundary layer may also cause excessive leaf temperatures that inhibit photosynthesis. At (very) high wind speed and low ambient air temperature, photosynthesis may be reduced due to below-optimal leaf temperatures and stomatal conductance, and to leaves rolling up [5, 16, 18]. Third, in some specific areas (e.g. coastal dunes, inland arid and semi-arid dunes), sand movement caused by strong wind is a common environmental agent affecting plants [20–24]. In these habitats, plants may face stress caused by soil losses or sand burial under wind erosion. In extreme cases, wind denudation may cause soil water loss, reduction in nutrient content or greater exposure of the upper roots of plants to high temperature [24–28]. In contrast, moderate sand burial may improve soil water and nutrient conditions and moderate the temperature fluctuation around plant roots, which may benefit plant growth [29].

Plants have developed various adaptations to cope with strong wind exposure, e.g. through trait variation in their branches [30] and leaves [31], biomass allocation [32], root structure [33], and/or stem mechanical properties [18, 34]. However, these traits of different plant organs are subject to trade-offs (e.g. carbon distribution) or coordination (e.g. allometric relationships). Therefore, it is hard from most previous studies focusing on particular plant organs and particular structural, morphological, and/or physiological traits, to infer whole-plant strategies in terms of adaptive response to wind. Few previous studies have compared wind responses among several species, even though plant species are known to vary in their capacity to resist wind, and to recover from or offset the effects of wind damage [35]. In addition, research fields study different aspects of plant response to wind. Forestry researchers tend to observe storm-damaged forest and often focus on the growth versus shade-tolerance trade-off of tree species [17, 36]. Agricultural research has focused on lodging resistance and yield-related traits [8, 15]. Thus, the scientific community is dispersed in the specific variables they measure in response to wind [6]. Research methods applied for wind manipulation also vary greatly, from field observation to field experiment to glasshouse controlled-environment experiment.

Two main experimental approaches have so far been used to impose wind disturbance effects on plants. One is artificially creating mechanical perturbation by shaking and brushing plants [5, 12, 19, 37–41], which has focused on the mechanical perturbation caused by wind and has been conducted mostly in controlled environments in glasshouses in the absence of wind. Another has been the use of wind tunnels or electric fans to enhance wind force [5, 16, 18, 32, 42, 43]. These methods depend on power supply and indoor facilities, which makes them hard to be widely applied under field conditions. Because of these limitations, we still understand poorly whether there are general syndromes of whole-plant response to wind exposure across organs, i.e. wind-coping strategies, or whether responses to wind are idiosyncratic among species without consistent responses among organs and their various traits.

Here we aimed to overcome the above limitations in a unique field experiment in order to test the null hypothesis that there are common whole-plant wind responses among different plant species. We assigned four contrasting vascular species to three treatments (reducing wind velocity, ambient wind velocity, enhancing wind velocity) in the steppe area of north China. To increase wind velocity we employed a novel methodology using connectivity modifiers (baffles) to converge wind. Connectivity modifiers, a patch-scale manipulation, can effectively change the size of connected pathways for wind or water under field conditions without directly affecting other abiotic and biotic factors. This methodology was previously used in some studies to collect foliar litter and seeds [44, 45], but has, to our knowledge, not been used to study wind effects on living plants. This methodology allows the wind regimes in this study to reflect the real environmental conditions that plants may encounter outdoors, where wind stress or disturbance may vary with weather conditions and the local properties of the terrain.

## Material and methods

### Study site

The experiment was carried out at the Ordos Sandland Ecological Station of the Chinese Academy of Science (OSES, 39° 29′ N, 110° 11′ E, 1296 m a.s.l.), located in the northeastern Mu Us Sandland in Inner Mongolia, China. Mean annual precipitation is 350 mm, with inter-annual fluctuations from 161 to 664 mm. Mean annual temperatures range from 5.0 to 8.5 °C, and mean potential evaporation is 2300 mm (OSES weather station, 2005–2015). On average 140 days per year have maximum wind speed > 6 bft (> 10.8 m/s), and the wind predominantly comes from the north-west (Additional file 1: Fig. S1). During the study year (2017), the total precipitation was 312.6 mm, most of which (280.4 mm) fell during the experimental period in the growing season (May to October; Additional file 1: Fig. S2A). Monthly mean wind velocities ranged from 1.6 to 2.31 m/s and maximum wind velocity (14.4 m/s) were reached in May (Additional file 1: Fig. S2B, C; all the wind data were collected from the OSES weather station at the standard 10 m height). The distribution of wind direction of maximum wind speed was consistent with these data (Additional file 1: Fig. S2D). The landscape in this area is characterized by mobile, semi-fixed and fixed sand dunes. As water availability is low, the area is dominated by steppe or desert vegetation with low height and sparse cover.

### Plant materials

Two predominant shrubs, i.e. *Artemisia ordosica* Krasch (Asteraceae), *Caragana intermedia* Kuang (Fabaceae) and two locally common herbs, i.e. *Agriophyllum squarrosum* (L.) Moq. (Chenopodiaceae) and *Salsola ruthenica* Iljin (Chenopodiaceae), were selected for the experiment. *A. ordosica* is a dominant shrub in (semi-)fixed sand dunes, approximately 0.5–1.0 m tall with plumose, linearly lobate leaves. Its lateral roots are mainly distributed in the upper 30 cm of the sand soil profile, while its primary roots may reach 1–3 m deep [27]. *C. intermedia* is a deciduous pinnate-leaved shrub approximately 1.5–2 m tall, widely used to mitigate desertification in north China [46]. *A. squarrosum* and *S. ruthenica* are annual herbs, up to 1 m tall, with erect stems and basal side-shoots. *A. squarrosum* is an important pioneer on moving and semi-fixed sand dunes, widely distributed in arid and semiarid regions of central Asia [47, 48] and widely naturalized in many other parts of the world.

Seeds of the four species were collected near OSES in 2015. On 16 September 2016, *A. ordosica* and *C. intermedia* seeds were germinated on wet filter paper; on 23 September 2016 the seedlings were transplanted into fabric bags (10 cm diameter, 15 cm height) and grown in the greenhouse of OSES through the winter. On 18 May 2017, 18 seedlings of each species with similar height (28.5 ± 4.07 cm for *C. intermedia* and 10.74 ± 0.89 cm for *A. ordosica*) and stem base diameter (1.91 ± 0.42 mm for *C. intermedia* and 4.00 ± 0.86 cm for *A. ordosica*,) were chosen and randomly transplanted into a 50 L (0.4 m diameter, 0.4 m high) pot filled with sand without drainage holes in the bottom. On 15 May 2017, *A. squarrosum* and *S. ruthenica* seeds were germinated on wet filter paper. After 16 days, 18 seedlings per species were transplanted into the pots (see above). All pots were buried in the quadrats of the three different treatments on 19 May, 2017. During the experimental period, all plants received 2 L of water once a week and 1 L of 1 g/L nutrient solution (Peters Professional 20-20-20 General Purpose, the Scotts Company, Ohio, USA; N:P:K = 1:0.83:0.44, plus microelements) once a month.

### Experimental design

**Fig. 1:**
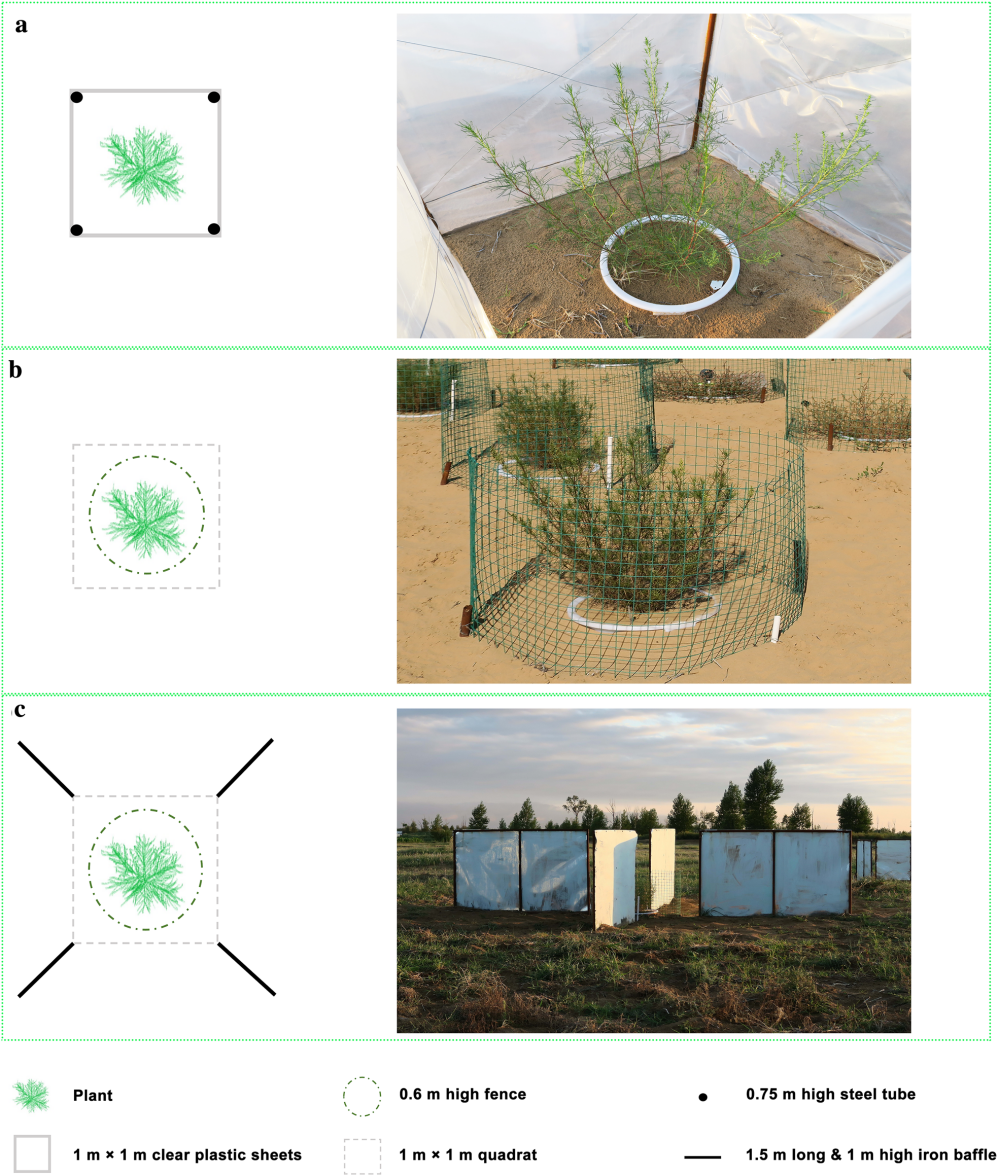
Experimental design and images of wind treatments. **a** The Decreased wind velocity treatment was implemented through a wind shield, which was 1 m × 1 m × 0.75 m (0.75 m high) and built with four steel tubes and transparent plastic sheets. The plastic chambers did not have a roof, so that ambient air could mix with that in the cubicles freely. **b** There was no additional treatment for the ambient treatment, apart from a large-mesh, 0.6 m high fence to avoid herbivory by wild rabbits (In a pilot test, the fence had a negligible influence on wind velocity). **c** The Increased wind velocity treatment was implemented through experimental wind baffles. We placed 1.5 m long and 1 m high iron sheets at each side directed towards the North-East, North-West, South-East and South-West, respectively from each pot, to converge and increase wind velocity. These baffles were at 0.7 m distance from the plant pot to avoid a shading effect

**Fig. 2:**
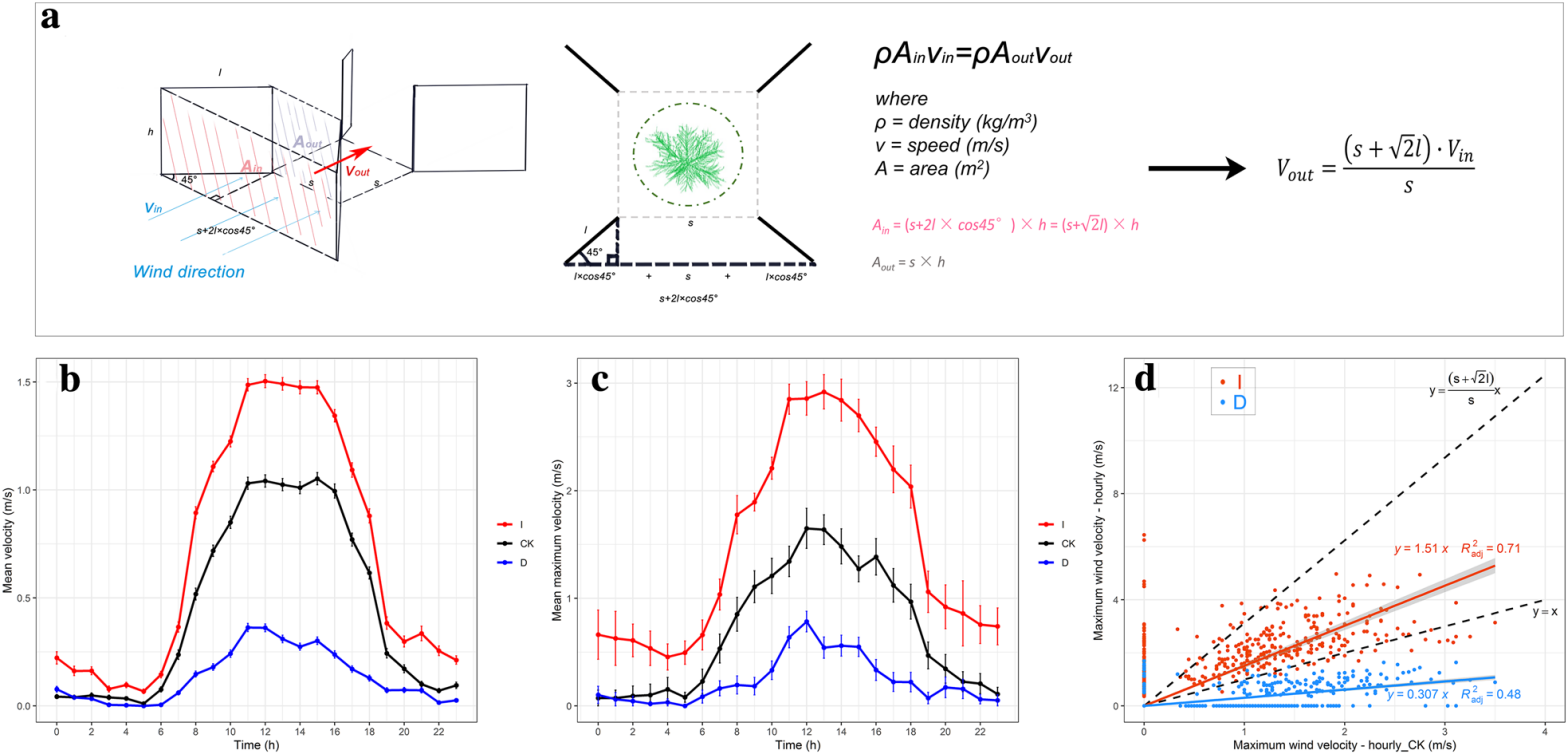
Diagrammatic sketch of wind velocity increasing methodology and effects of wind treatments. **a** Wind velocity increasing method. *A*_*in*_ is the interface area of the wind flow coming in and *A*_*out*_ is the interface area of the wind flow going out. *V*_*in*_ is the initial in-coming wind velocity and *V*_*out*_ is the wind velocity of air flowing out. *s* is the length of the short side of the wind funnel, and *l* is the length of the wind baffle. **b** Diurnal mean wind velocity pattern (mean ± SE) over 24 h under wind treatments throughout the experimental period. **c** Diurnal maximum wind velocity pattern (mean ± SE) over 24 h under wind treatments throughout the experimental period. **d** Effects of wind treatments. The black dashed line with the equation ($\text{y}=\frac{(s+\sqrt{2}l)}{s}x$) represents the ideal wind velocity that could be reached by acceleration. The black dashed line with the equation (*y* = *x*) represents the wind velocity in the ambient treatment (CK). Red dots and line represent the relationship between the temporally matched hourly maximum wind velocity in I and CK. Blue dots and line represent the relationship between the temporally matched hourly maximum wind velocity in D and CK. Regression equations and *R*^2^ are given. D means decreased wind velocity treatment, CK means ambient wind velocity treatment, and I means increased wind velocity treatment

We imposed three different wind treatments on the four plant species: decreased wind velocity (D), ambient wind velocity (CK), and increased wind velocity (I); see Fig. 1 for methodological details. In brief, treatment D was implemented through a wind shield (Fig. 1a). The design of using transparent plastic sheets was intended to minimize the influence of the wind shield on the light regime. Treatment I was implemented through artificial air baffles that funneled the ambient wind towards the target plant (Fig. 1c). To the best of our knowledge, this represents a new method for wind enhancement with minimal experimental artefact (see under “Results”). The wind velocity increasing method was based on an ideal model (Fig. 2a) coming from the fluid continuity equation:

1 $$ \rho A_{1} V_{1} = \rho A_{2} V_{2} . $$

where *ρ* was a constant representing the density of air flow. *A*_1_ was the interface area of the wind flow coming in and *A*_2_ was the interface area of the wind flow going out. *V*_1_ was the initial wind velocity when air flow came in and *V*_2_ was the wind velocity when air flow went out. In the ideal model, air should flow through a closed tunnel. The basic continuity equation, states that the amount of air flowing in on one side must equal the air flowing out on the other side. In this case, we could change the velocity of air flow by changing the ratio of *A*_1_ and *A*_2_. In our design, we used air baffles to create an air tunnel that had a larger area where the wind flow entered than the area where the wind flow exited. According to the continuity equation,

2 $$ V_{out} = (s + \sqrt 2 l)V_{in} /s $$

where *V*_*out*_ represents the wind velocity at the exit point of the wind funnel, and *V*_*in*_ is the initial wind velocity before treatment. *s* is the length of the short side of the wind funnel, *l* the length of the wind baffle. However, the above formula assumes a closed funnel at the top. As our wind funnel was open at the top, the actual wind velocity at the exit point of the wind funnel should be smaller than *V*_*out*_. Increasing the baffle height would make the funnel closer to the sealed status, i.e. it could make the final wind velocity closer to *V*_*out*_.

Both treatments CK (Fig. 1b) and I had a large-mesh fence around the plant to protect it from herbivory. In total, there were 72 plants: 4 species × 3 treatments × 6 replicates (Fig. 1). The experimental site was very flat, and treatments were far away from each other and taller surrounding vegetation. For avoiding obstruction of the air flow to other treatments, the D section was laid out at the south-west, the CK section was set out at the east and I section at the west of the experimental site. During the experiment period, sand burial happened due to the increased wind velocity in treatment I despite the protection of the plastic pot. Extra sand was periodically removed from the pots using a brush when burials happened to minimize the influences of the sand burial on plants.

### Environment parameters measurements

Wind speed, air temperature and relative humidity were measured at 0.8 m above the ground (i.e. close to maximum plant height), using AZ9671 Anemometer Loggers (Shenzhen frank electronics co. LTD., China). The loggers recorded data with 90-s intervals. Because the AZ9671 Anemometer Loggers can only quantify air movement in one direction, we chose westerly as our main direction (due to prevailing strong wind coming from this direction; see Additional file 1: Fig. S1) and all the loggers were positioned toward the west during the whole experiment period. Soil volumetric water content and temperature were measured 10 cm below the soil surface in the middle of the buckets by Em50 series data loggers (Decagon Devices, Inc., USA). The loggers collected data at 2-min intervals. In each treatment section, three AZ9671 and Em50 loggers were installed in random positions. We collected data from 1 June to 7 September 2017. Due to the limited of the power supplies and data storage of AZ9671 loggers, we had to retrieve the loggers from the field for replacing batteries and download data regularly. Additionally, the anemometer had a high rate of malfunction in the rain. Thus we also avoid the continuously rainy days. In this case, the data set of wind speed, air temperature and relative humidity had some breaks in time during the whole experimental period, but we tried our best to make sure that the measurements covered most of the available time. The specifications of all the instruments used in this study can be found in Additional file 1: Table S1.

### Plant trait measurements

All plants were harvested within nine days from 7 September 2017.

Plant height, stem base diameter (SBD), crown traits, leaf traits and branching pattern were measured before harvest. Plant crown length (L) and width (W) were measured, and projected crown area (PCA) was calculated as

3 $$ {\text{PCA}} = 0.{25}\pi \, LW $$

For each plant, 15 fresh leaves were scanned and leaf length (LL) and leaf area (LA) were measured. The leaves were dried at 80 °C for 48 h, then weighed. Specific leaf area (SLA) was calculated as fresh leaf area per unit leaf dry mass.

The branching pattern was measured following Strahler method [49]. Numbers of first to third degree branches were counted, then overall bifurcation ratio (OBR) and stepwise bifurcation ratio (SBR) were calculated as

4 $$ {\text{OBR}} = \left( {N_{t} - N_{s} } \right)/\left( {N_{t} - N_{1} } \right) $$

5 $$ {\text{SBR}}_{{({\text{i}}:{\text{i}} + {1})}} = N_{i} /\left( {N_{i + 1} } \right) $$

where numbers of branches are represented by *N*_*t*_ for all segments, *N*_*s*_ for the highest-order branches, *N*_*1*_ for the first-degree branches, while N_i_ the branch number of *i* degree.

After harvest, each plant was divided into root and shoot. We measured root length (RL) of the longest root. For shoots, 15 cm long primary stem sections were separated from the bottom of the plant shoot for determination of mechanical properties.

Young’s modulus (*E*) of the main stems was measured with an electromechanical device (Type 5540; Instron, Norwood, MA, USA), applying the three-point bending technique [50]. *E* is a measure of stiffness of a material [7]. Vertically applied forces (F; N) and resulting deflections ($\delta$; m) were recorded. *E* was calculated as

6 $$ E = \left( {FL^{3} } \right)/48\delta I $$

where L is the length between the supports (m) and *I* the second moment of area (m^4^). *I* was calculated as

7 $$ I = (\pi r^{4} )/4 $$

where r = stem radius in m. This index is a measure of the geometric contribution to rigidity [7]. Flexural stiffness of stems is a measure of the rigidity of a material, calculated as the product of *E* and *I* (*EI*, N/m^2^; [7]).

Mechanical measurements of each plant were completed within 15 min after cutting. After these tests, all parts of the plants were dried at 80 °C for at least 72 h. Then, root and shoot biomass were weighed and root/shoot ratio was calculated. After weighing, the main stems used for mechanical measurements were ground by ball mill; stem lignin content (SLC) was determined as acid insoluble (Klason) lignin. Stem cellulose content (SCC) was determined colorimetrically with the anthrone reagent [51].

### Data analysis

Because the data set of wind speed, air temperature and relative humidity had breaks in time, only days with the completed data were chosen for the analysis. In total there were 31 completed days that covered the whole experimental period. Then, the average hour-by-hour values of each parameter in each treatment throughout this period were calculated. Based on the hourly maximum wind velocities in consecutive days, the hour-by-hour mean hourly maximum wind velocity was calculated. To evaluate the results of wind velocity changes, the hourly maximum wind velocity in the treatment CK and I, and Treatment CK and D were temporally matched, then the linear regression lines were generated by the formula ($y=ax$) in *R* software (v3.3.0, R core team, 2015). Because of the limitation of the AZ9671 Anemometer Logger, wind velocity data were collected from only one direction (west). The wind velocity data collected from the directions perpendicular to the logger were expected to be underestimates. For checking the accuracy of our wind velocity data, we used the wind data collected from OSES weather station as a reference. The OSES weather station recorded the hourly average wind direction during the whole experimental period. We first extracted the time intervals during which the wind came from the west (from 225° to 315°) during the experimental period. Then, we paired these time points with the data collected from the AZ9671 Anemometer Logger, extracting the time intervals each day when the wind was mainly coming from the west during our experimental period. By comparing the average hour-by-hour wind velocities between the westerly wind data set with those in the whole data set, we could evaluate the accuracy of our whole wind velocity data set collected from AZ9671 Anemometer Logger, and especially the robustness of the differences found among wind treatments. Two-way ANOVAs were used to analyze the (interactive) effects of different species and wind treatments on each plant trait. One-way ANOVA was used to test differences in plant traits among the three wind treatments for each species separately, followed by Tukey’s HSD tests for multiple comparisons. Data were log_10_(x + 1)-transformed if necessary to improve the equality of variance distributions among treatments. Statistical analyses were performed using SPSS18 (SPSS Inc., USA2009). A redundancy analysis was carried out in *R* software (v3.3.0, R core team, 2015) using the package Vegan to explore the covariance structure of the different trait responses to the wind treatments across the species comprehensively. The plant traits were used as the respective response variables. Then, the response matrix was standardized by a scale function. The explanatory matrix was defined by the plant species and wind treatments. For testing the significance of the variation in response matrix explained by explanatory variables, a Monte Carlo permutation test was used (Anova function with permutation options, permutations = 999). The adjusted *R*^2^ was computed, after the permutation test.

## Results

### Environmental variables in different wind treatments

The wind shields (treatment D) reduced daily ambient wind velocity by 71% and maximum wind velocity by 67% on average (Fig. 2b, c). It increased ambient air temperature on average by 2.1 °C, and reduced ambient relative humidity by 3%, ambient soil temperature by 0.4 °C and ambient soil volumetric water content by 14% on average (Additional file 1: Fig. S3). The experimental wind baffles (treatment I) increased daily ambient wind velocity by 56% and maximum wind velocity by 114%, decreased ambient air temperature by 0.6 °C and ambient soil temperature by 1.7 °C on average, while they increased ambient relative humidity and soil volumetric water content each by 2% (Fig. 2b, c, Additional file 1: Fig. S3). The average hour-by-hour wind velocity between the westerly wind data set and the whole data set (Additional file 1: Fig. S4) showed similar patterns when comparing the mean wind velocities among different wind treatments. This means that, although the limitation of the AZ9671 Anemometer Logger caused some inaccuracy in absolute wind velocity values, the whole data set still reflected the real wind patterns in the different treatments during our experimental period.

The temporally matched relationship between the hourly maximum wind velocity in the CK and I showed that our wind funnel design had potential to accelerate wind flow reaching the velocity calculated by the ideal model (Fig. 2d). According to the regression equation *y* = 1.51*x*, the actual accelerated hourly maximum wind velocity of our design can be predicted by the hourly maximum wind velocity in the ambient condition. The temporally matched relationship between the hourly maximum wind velocity in the CK and D also reflected the effect of the wind breaks on decreasing wind velocity. Another regression equation *y* = 0.307x was generated which could be used to estimate the maximum wind velocity in the treatment D.

### Commonalities in responses among the species to wind treatments

All plant traits showed significant differences among species, while 10 out of 19 traits showed significant wind effects and nine out of 19 traits significant interactions of species and wind treatment (Table 1). Across species, wind velocity significantly affected plant height, PCA, SBD, RL, SBR_(1:2)_, LL, total and shoot biomass, root/shoot ratio, and *E*. There were significant species by treatment effects on PCA, SBD, OBR, SBR_(1:2)_, root/shoot ratio, SCC, *I* and *E* (Table 1).

**Table 1:** Effects of species and wind velocity treatments, and their interactions on plant traits

Plant traits	Species (S)		Wind treatments (T)		S × T	
	F	P	F	P	F	P
Morphology						
Height	43.16	*< 0.001*	20.06	*< 0.001*	1.31	0.268
Projected crown area	146.12	*< 0.001*	32.33	*< 0.001*	3.09	*0.012*
Stem base diameter	204.97	*< 0.001*	10.51	*< 0.001*	3.00	*0.013*
Root length	71.48	*< 0.001*	10.35	*< 0.001*	1.59	0.167
Overall bifurcation ratio	10.52	*< 0.001*	2.26	0.113	3.35	*0.007*
Stepwise bifurcation ratio_(1:2)_	13.18	*< 0.001*	3.29	*0.044*	3.47	*0.005*
Leaf length	359.76	*< 0.001*	4.81	*0.012*	1.03	0.413
Leaf area	51.88	*< 0.001*	0.73	0.485	0.29	0.939
Specific leaf area	248.26	*< 0.001*	0.08	0.921	0.30	0.937
Biomass and allocation						
Total biomass	171.96	*< 0.001*	9.60	*< 0.001*	0.35	0.908
Shoot biomass	246.47	*< 0.001*	10.67	*< 0.001*	0.17	0.846
Root biomass	19.01	*< 0.001*	2.63	0.080	0.77	0.599
Root/shoot ratio	61.17	*< 0.001*	10.82	*< 0.001*	7.40	*< 0.001*
Mechanical properties						
Stem lignin content	11.71	*< 0.001*	0.38	0.687	0.72	0.637
Stem cellulose content	5.41	*0.002*	1.67	0.196	7.90	*< 0.001*
Lignin/cellulose ratio	11.16	*< 0.001*	0.7	0.500	3.11	*0.010*
Second moment of area (*I*)	16.57	*< 0.001*	1.51	0.231	3.79	*0.003*
Young’s modulus (*E*)	10.93	*< 0.001*	10.63	*< 0.001*	4.52	*0.001*
Flexural stiffness (*EI*)	14.68	*< 0.001*	3.09	0.054	0.69	0.660

Italic type indicates significant differences at p < 0.05

**Fig. 3:**
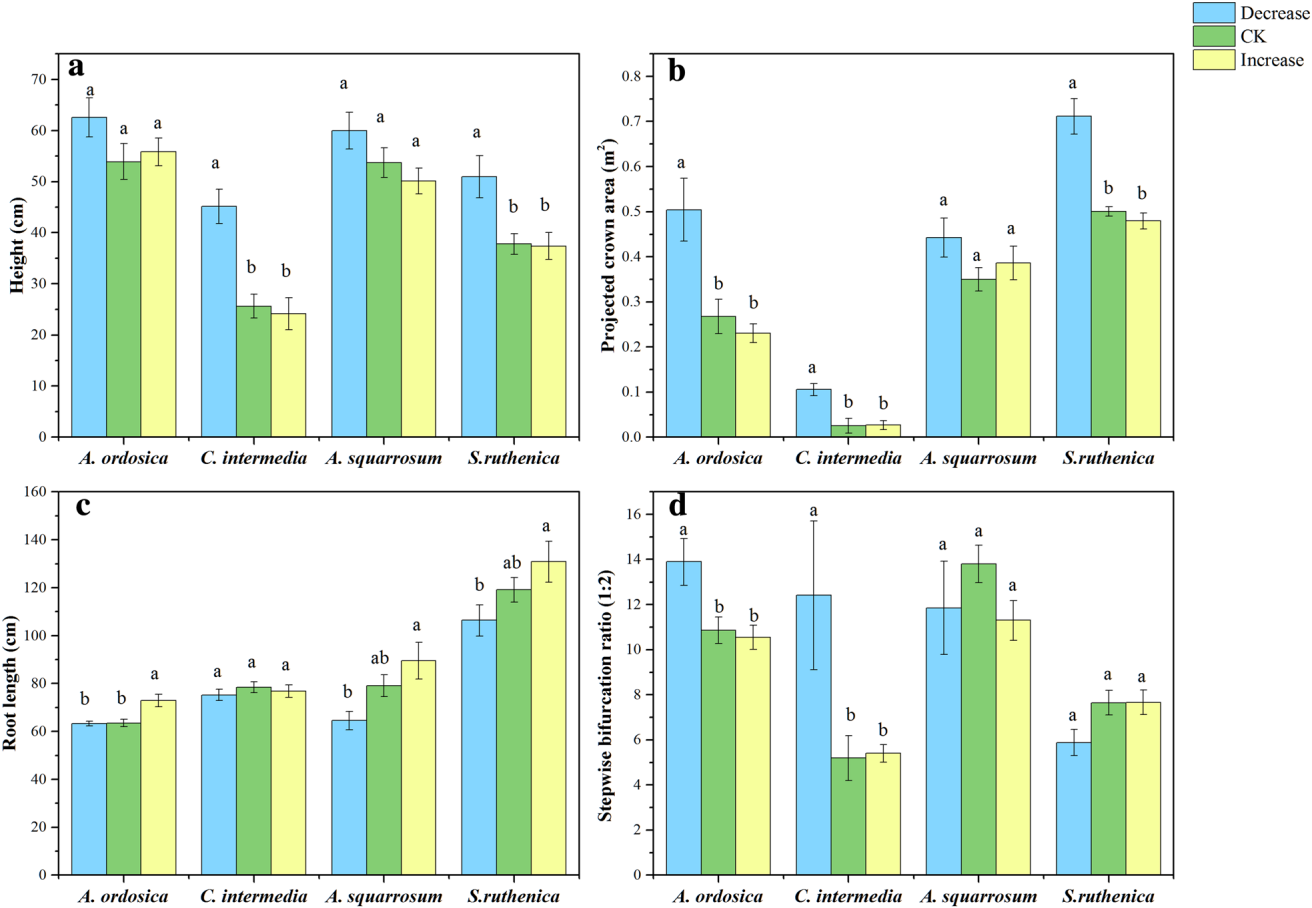
Plant size and shape traits of four plant species under different wind treatments. Plant height (**a**), projected crown area (**b**), root length (**c**) and STEPWISE bifurcation ratio_(1:2)_ (**d**). Decrease, CK (ambient) and Increase refer to wind velocity treatments. Different lowercase letters indicate significant differences among treatments at p < 0.05. The error bars are plotted by means ± SE

**Fig. 4:**
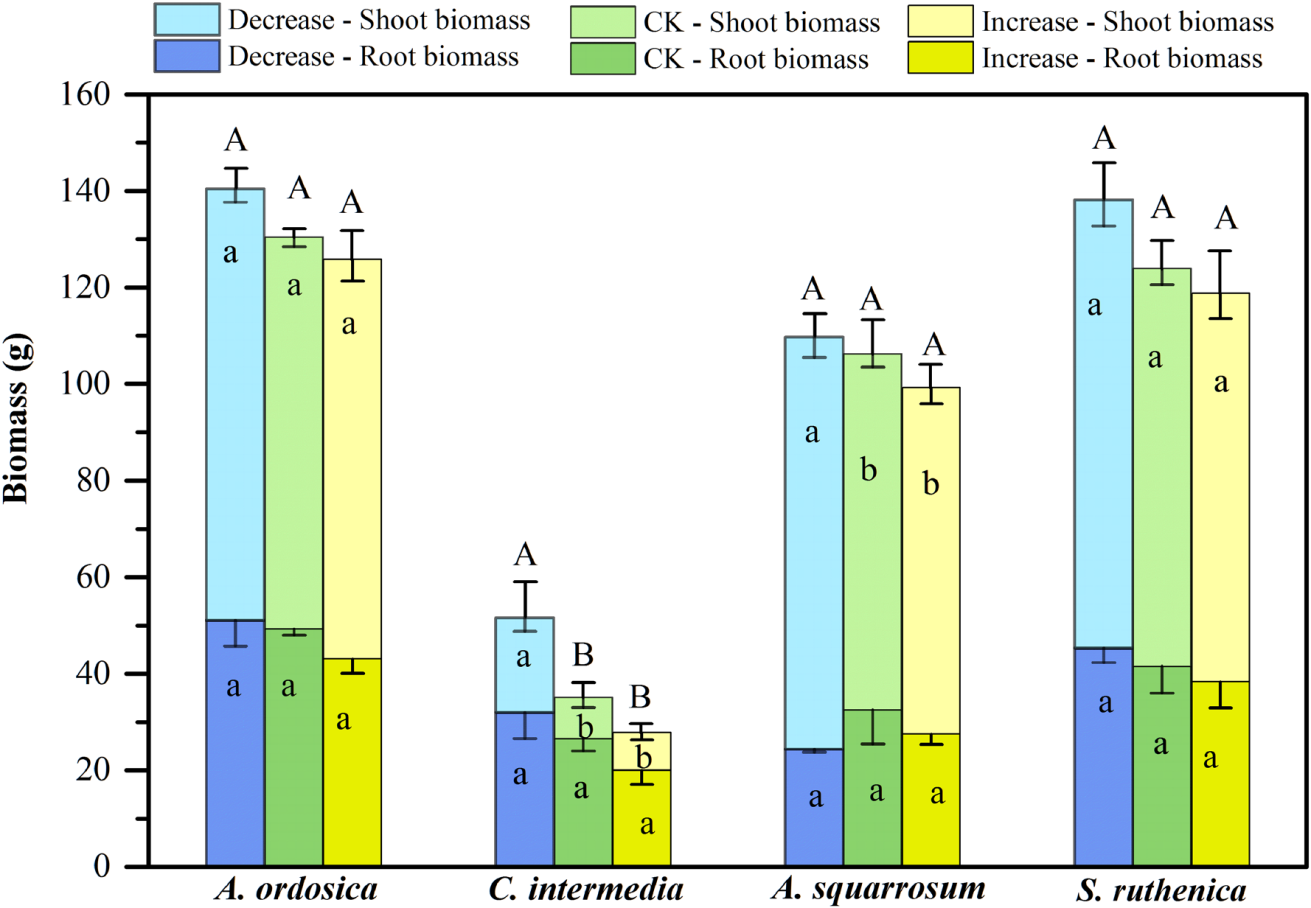
Plant biomass and its allocation in four plant species under different wind treatments. Decrease, CK (ambient) and increase refer to wind velocity treatments. Different uppercase and lowercase letters indicate significant differences among treatments at p < 0.05. The error bars are plotted by means ± SE

**Fig. 5:**
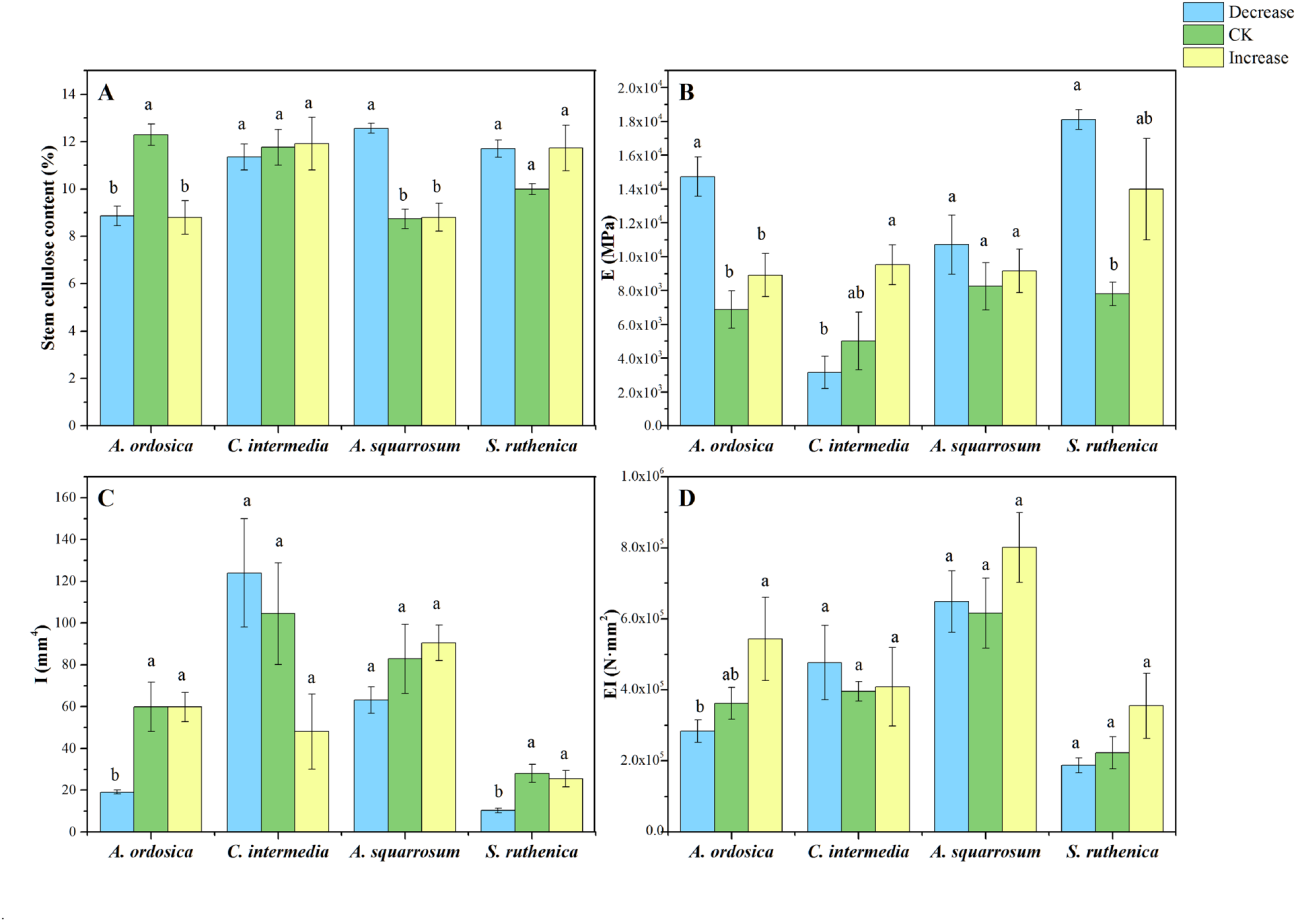
Shoot property traits linked to wind resistance in four plant species under different wind treatments: **a** Stem cellulose content, **b** Young’s modulus (*E*), **c** Second moment of area (*I*), and **d** Flexural stiffness (*EI*). Decrease, CK (ambient) and Increase refer to wind velocity treatments. Different lowercase letters indicate significant differences among treatments at p < 0.05. The error bars are plotted by means ± SE

Among species, common responses mainly showed as decreases in size-related traits and increases in wind-resistance abilities. Plants tended to be shorter, have smaller PCA, SBR_(1:2)_, and lower biomass in response to increasing wind velocity (Table 2, Figs. 3, 4). Height of *C. intermedia* and *S. ruthenica* decreased with increasing wind velocity (Fig. 3a). PCA of *A. ordosica*, *C. intermedia*, and *S. ruthenica* decreased with increasing wind velocity (Fig. 3b). OBR and SBR_(1:2)_ of *A. ordosica* and *C. intermedia* were markedly influenced by the wind treatments (Table 2), being lower in CK and I than in D (Fig. 3d, Additional file 1: Fig. S5E)*.* Increased wind velocity decreased total biomass of *C. intermedia*, and shoot biomass of *C. intermedia* and *A. squarrosum* (Table 2, Fig. 4). RL, SBD and *I* tended to increase with increased wind velocity (Table 2, Figs. 3, 5, and Additional file 1: Fig. S5). RL of *A. squarrosum* and *S. ruthenica* increased steadily from D via CK to I while *A. ordosica* only showed an increase from CK to I and *C. intermedia* showed no response at all (Table 2, Fig. 3c). *I* of *A. ordosica* and *S. ruthenica* was higher in treatments CK and I than in D (Table 2, Fig. 5c). Stem base diameter of *A. ordosica* and *A. squarrosum* increased in CK and I compared to treatment D (Table 2, Additional file 1: Fig. S5F).

**Table 2:** Effects of wind velocity treatments on plant traits of *A. ordosica*, *C. intermedia*, *A. squarrosum*, and *S. ruthenica* based on one-way ANOVAs

Plant traits	*A. ordosica*		*C. intermedia*		*A. squarrosum*		*S. ruthenica*	
	F	P	F	P	F	P	F	P
Morphology								
Height	1.82	0.201	15.55	*< 0.001*	2.51	0.131	6.40	*0.010*
Projected crown area	10.97	*< 0.001*	10.57	*0.003*	1.64	0.238	25.24	*< 0.001*
Stem base diameter	5.50	*0.016*	0.59	0.567	4.11	*0.038*	2.13	0.155
Root length	8.57	*< 0.001*	0.50	0.617	5.48	*0.017*	3.00	0.085
Overall bifurcation ratio	6.03	*0.012*	4.71	*0.026*	1.29	0.304	16.36	*< 0.001*
Stepwise bifurcation ratio_(1:2)_	6.51	*< 0.001*	4.23	*0.035*	0.91	0.423	3.38	0.062
Leaf length	2.97	0.082	5.51	*0.016*	0.30	0.747	4.40	*0.031*
Leaf area	0.06	0.942	4.52	*0.029*	3.79	*0.047*	0.04	0.966
Specific leaf area	1.15	0.342	0.51	0.610	0.01	0.992	0.19	0.826
Biomass and allocation								
Total biomass	3.04	0.078	6.94	*0.007*	0.93	0.415	1.85	0.192
Shoot biomass	1.22	0.323	9.22	*0.002*	4.45	*0.030*	1.99	0.171
Root biomass	1.39	0.279	2.53	0.113	0.85	0.448	0.60	0.561
Root/shoot ratio	2.50	0.121	4.52	*0.049*	5.06	*0.021*	0.10	0.907
Mechanical properties								
Stem lignin content	0.59	0.566	0.01	0.993	0.14	0.870	1.51	0.252
Stem cellulose content	13.72	*< 0.001*	0.12	0.885	25.94	*< 0.001*	2.68	0.101
Lignin/cellulose ratio	4.74	*0.025*	0.02	0.984	4.52	*0.029*	0.68	0.522
Second moment of area (*I*)	8.80	*< 0.001*	2.54	0.124	1.56	0.243	6.43	*0.014*
Young’s modulus (*E*)	11.83	*< 0.001*	5.57	*0.021*	0.70	0.513	6.93	*0.011*
Flexural stiffness (*EI*)	3.70	*0.049*	0.27	0.770	1.10	0.359	1.86	0.201

Italic type indicates significant effects at p < 0.05

### Idiosyncratic responses among species to wind treatments

In contrast to the common responses to wind treatments for size-related traits, the plants showed idiosyncratic responses among species for some traits (Additional file 1: Fig. S6). Responses of mechanical properties showed large diversity. Wind decreased *E* in *A. ordosica* and *S. ruthenica*. *E* of *A. ordosica* decreased markedly in CK and I as compared to D, which resulted in an increase of *EI*. In *S. ruthenica*, *E* decreased in CK. However, the wind treatments had no significant influence on *E* of *A. squarrosum* and increased *E* of *C. intermedia*. Except for *A. ordosica*, *EI* of the other three species was not significantly influenced by the wind treatments. Moreover, SCC of *A. ordosica* was higher in CK than in D, while in *A. squarrosum* it was lower in CK and I than in D (Fig. 5).

Leaf traits also responded in a species-specific manner to the wind treatments. Leaf length of *C. intermedia* and *S. ruthenica* and leaf area of *C. intermedia* and *A. squarrosum* were influenced by wind treatments (Table 2). The leaves of *S. ruthenica* were shorter in CK and I as compared to D (Additional file 1: Fig. S5A). Leaf area of *C. intermedia* was markedly reduced in CK as compared to D, and leaf area of *A. squarrosum* was lower in CK and I than in D (Additional file 1: Fig. S5C).

## Discussion

To our knowledge, this is the first experimental study to simultaneously compare effects of both decreased and increased wind speed on plant performance with ambient wind speed under field conditions. Our wind funneling treatment has added a new experimental method to increase wind speed under field conditions without some of the experimental artefacts on other environmental factors associated with treatments using wind tunnels and fans. In particular, our field wind-funneling baffles continuously increased wind speed proportionally (increased daily ambient wind velocity by 56% and maximum wind velocity by 114%, Fig. 2) to ambient wind speed. These treatments were effective in altering wind speed with the intended magnitude of reduction or increase throughout the day across the growing season (Fig. 2). This new experimental design has been able to reveal how four morphologically different steppe plant species, including two shrubs and two forbs, responded in apparently adaptive ways to both reduced and increased wind speed. Overall, these responses showed both common patterns among these species, especially for traits related to plant and organ size, and idiosyncratic patterns, which were seen mostly for traits related to shoot and leaf properties. Correspondingly, we will first discuss the commonalities in trait response among the species, followed by a focus on the traits that showed idiosyncratic responses among species. We will also discuss the possible confounding influences of the treatments on plant performance via microclimate effects on leaf boundary layer and gas exchange, which we did not measure. Finally we will discuss the effects of our wind treatments and their future application.

### Common response patterns of plant size related traits among species

In general, plants can adapt to wind stress at the whole-plant level (i.e. besides possible effects via photosynthesis and transpiration) either by reducing the mechanical stress through lower height and smaller crown; and/or by increasing the resistance abilities via increasing stem base diameter, root length, and/or changing mechanical properties. Our results showed that across species, wind velocity significantly affected plant shoot biomass and morphology traits at the whole-plant level, particularly plant height, projected crown area, stepwise bifurcation ratio, root length and stem diameter. Increased wind velocity had negative effects on plant height, PCA and total biomass across all species. In the two shrub species (*A. ordosica* and *C. intermedia*), SBR_(1:2)_ also showed a negative trend with the increase in wind velocity. In contrast, there were positive trends with root length in all species. SBR_(1:2)_ indicates directly branching conditions in the current year [52], and decreased of SBR_(1:2)_ means that plants reduce the numbers and densities of branches. Together with a decrease of PCA, lower SBR_(1:2)_ will reduce the wind drag to whole plants. These results are consistent with most previous studies on single plant species, where plant height usually decreased and stem base diameter increased at high wind velocity [12, 14, 15], plant biomass and PCA decreased [4, 10] and root/shoot ratio increased [9, 16]. In summary, across species, plants will reduce the mechanical stress caused by wind through smaller stature and increase the physical resistance against wind through deeper and coarser roots.

Treatment D had stronger effects on size-related traits than treatment I, such as on PCA in *A. ordosica* and *C. intermedia*, height of *C. intermedia* and *S. ruthenica*, and shoot biomass of *C. intermedia* and *A. squarrosum*. How can we explain this? Firstly, on an evolutionary time scale, windy conditions have probably been more common than still conditions [53–55]. The monthly mean wind speeds during our experimental period were below 2.5 m/s, i.e. were lower than those in most other parts of the Inner Mongolian Plateau region. Regional ecotypes may thus be adapted to higher wind exposure. Secondly, there was a 71% reduction and a 56% increase of daily wind velocity and a 67% reduction and a 114% increase of daily maximum wind velocity compared to the control treatment. This difference in treatment effect may have led to different effect sizes of response. By adjusting the height and length of the artificial air baffles in treatment I, we should be able to adjust the wind speed increase. Longer baffles will be needed to obtain stronger wind forces to cover the full range of wind velocities different plants may experience not only in steppe but also in other ecosystems.

### Idiosyncratic shoot trait response patterns among species

Mechanical perturbation caused by wind in nature has long been examined [12, 16], and is known to change the flexibility and rigidity of plant stems or petioles [9, 19, 56]. Plants may either develop flexible stems for reducing the stress imposed by wind drag force; or stiff stems to resist wind [19, 57, 58]; and there may be trade-offs between these two aspects. Therefore, the response patterns of shoot traits, especially mechanical properties, to wind velocity is expected to be idiosyncratic among species.

In this experiment, wind significantly increased the second moment of area (*I*) of *A. ordosica* and decreased Young’s modulus (*E*) of stem of *A. ordosica* and *S. ruthenica*, resulting in a significant increase of Flexural stiffness (*EI*); while wind did not significantly affect *EI* in the other three species. This is consistent with previous studies [59, 60], in which *EI* remained constant or increased. Yet, a constant *EI* value does not mean that the mechanical properties do not change, because *EI* depends on both *E* and *I*. Actually, wind significantly increased *E* in *C. intermedia*, and *I* in *S. ruthenica* in our experiment; while wind did not change *E*, *I* or *EI* in *A. squarrosum*. Thus, both *A. ordosica* and *S. ruthenica* tended to become more flexible under strong wind stress while *C. intermedia* shoots tended to increase their rigidity.

The change in *EI* in this experiment is likely a result of the change in stem base diameter, change in symmetry and/or amounts of chemical compounds such as plant stem cellulose content, all of which may have influenced the flexibility of the stem [60]. SBD is tightly correlated with *I*, the rigidity index [7]. In *A. ordosica*, the increase of SBD in treatment I corresponded with the increase of *I*, and thereby increased *EI*. But the increase of SBD in *A. squarrosum* did not coincide with changes in mechanical properties, which may be attributed to the cancellation effect of a reduction of cellulose content.

Most of the time, the effects of wind on plants are modified by other environmental factors. The responses to wind are specifically modified by shading, the nutritional status of plants, by soil water and inherent plant traits, such as clonality [16, 19, 32, 61]. These factors may also enhance the idiosyncrasy of shoot trait response patterns to wind among species. Additionally, in the Mu Us Sandland, Aeolian sand displacement is an important environmental factor. Wind denudation and sand burial are the two main sand mobility processes that will also modify the effects of wind on plants (see “Background”). For example, the root to shoot ratio of *A. ordosica* seedlings was found to increase while the height and stem diameter decreased under wind denudation of the soil surface [27, 62]. The *E*, *I* and *EI* of *C. intermedia* were found to decrease with sand burial [23]. These traits were also influenced by our experimental wind perturbation. Thus, plant responses to sand movement may also modify plant wind resistance traits. Although the influences of windblown sand on plants were minimized in our experiment, it is still a very interesting question to address in further studies. In-depth studies are needed to focus on the effects of confounded factors related to climate, soil properties, Aaeolian sediment and inherent plant traits on response patterns to wind.

### Indirect wind effects on species’ performance through changing microclimate

Under field conditions, besides affecting plant performance through changing their leaf traits, wind can also influence photosynthesis and transpiration through changing the microclimate. Different from branches and trunks, leaves are highly flexible, and only very strong wind gusts could cause significant damage, such as being torn, shredded or pulled off the branches [63]. Under less extreme wind condition, many previous studies on single species found that wind reduced the number of leaves, leaf area and leaf dry mass, with an increase of leaf thickness [5, 7, 17–19, 27]. Our results showed that, across plant species, wind significantly affected leaf length only, but had no significant effects on leaf area or SLA. The leaf traits measured also showed very different response patterns to wind.

Wind speed plays an important role in the microclimate around a leaf attached to the plant. Increased wind velocity could result in lower leaf boundary layer conductance which can cool the leaves and improve gas exchange at higher temperatures [64, 65]. At low ambient air temperature, photosynthesis may be reduced due to below-optimal leaf temperatures and stomatal conductance [5, 16, 18].

In contrast, at low wind speed, a thicker leaf boundary layer may cause high leaf temperatures and inhibit photosynthesis. In treatment D, the peak wind velocity at noon was on average less than 0.5 m/s (Fig. 2b). Under such conditions the already high air temperatures (Additional file 1: Fig. S3) will be amplified by the boundary layer potentially risking acute heat damage [63]. The negative effects of low wind treatments on leaf performance through excessive leaf temperatures may explain why total biomass did not increase in three species out of four (i.e. except *C. intermedia*) under our low wind treatment. Smaller or narrower leaves are more suited to withstand high air temperature due to better convective dissipation [63]. This could explain why the leaf area of *A. ordosica* and *S. ruthenica* did not increase significantly under low wind treatment, even though leaf area tended to increase at low wind condition overall. At the same ambient air temperature, pinnate leaves dissipate heat more effectively than simple ones [63]. *C. intermedia* has pinnate leaves, which should help it to cope with high air temperature. Trichomes can also help leaves to substantial reduce sunlight absorption, which can reduce the damage due to low wind and high temperature [63]. There were 10–50 trichomes/mm^2^ on the leaves of *A. squarrosum* and more than 50 trichomes/mm^2^ on the leaves of *C. intermedia* [66]. This may explain why leaf area of *C. intermedia* and *A. squarrosum* could increase significantly under low wind velocity.

### Effects of the wind treatments and its future application

Our new field wind-funneling design (I) showed high potential in synchronously increasing the wind velocity in the treatment area roughly in proportion to the ambient wind velocity. Through the four funnel entrances in four different directions, the wind can be accelerated in different directions, which ensures that with the change of wind direction, the wind flowing through the funnel can be continuously accelerated in an effective way. According to formula *V*_*out*_ = (*s*+$\sqrt{2}$*l*)*V*_*in*_/*s*, it is possible to adjust the wind speed increase by adjusting the length of the artificial air baffles. Wind acceleration effects can be estimated according to the quadrat length and the length of baffles. In another ongoing experiment of ours, we are also attempting to use this design at community scale to detect the response of *A. ordosica* community to the increased wind speed (Additional file 1: Fig. S7A). In that experiment, 4 m × 4 m quadrats were set out and four 5 m long and 1.2 m high baffles were used to increase the wind velocity. The results showed that the wind funnel design also had potential to be used at community scale (Additional file 1: Fig. S7B–D). The experimental wind baffles (treatment I) increased daily ambient wind velocity by 47% and maximum wind velocity by 130%, while decreasing ambient air temperature by 0.7 °C on average.

The height of the baffle would not affect wind velocity increase in our ideal model, while increasing the baffle height would make the funnel closer to the sealed status, which should make the results for the wind increase treatment closer to those calculated by the model. Thus, we suggest that the height of the baffle should also be considered when building new wind funnels for getting better acceleration results. Additionally, according to the fluid continuity equation, the border of the exit side of the wind funnel has the shortest width, which means that the wind velocity peak appears at this point. Because the quadrat was set at 1 m × 1 m in our experiment, i.e. a small area, the wind velocity may not vary much within the quadrat. In larger quadrats wind might decline through the quadrat in the wind flow direction. Such a gradient in the wind velocity should also be considered in future studies.

Our wind shield design (D), like many other wind reduction designs, was effective in decreasing wind velocity. However, the use of wind shields could cause two main confounding effects: light shielding and heat trapping. The choice of using transparent plastic sheets was based on the intention to minimize the influence of the wind shield on the light regime. However, we are aware that the plastic sheets used in treatment D could alter light quality somewhat by intercepting or reflecting certain wavelength bands more than others. Especially the red: far-red ratio could have been affected, which is known to affect several plant performance parameters [67, 68]. Also, the reflection of the red and far-red part of the light spectrum might have caused a minor increase of air temperature. In future experiments of this kind, a material should be used that causes minor and evenly distributed interception and reflection of the whole range of plant-relevant wavelengths and the actual interception should be measured. Our wind decreasing treatment increase the air temperature (especially around noon) by, on average, 1.7 °C; even though the rationale of this treatment was to decrease wind velocity simultaneously with wind velocity enhancing methods under field conditions in a way that had minimal influences on the other environmental factors. This drawback may be overcome by adjusting the fencing method. For example, we also tried a slightly different design with a larger fencing area and distance between quadrat and fences (Additional file 1: Fig. S7A); this design seemed to have less influence on the air temperature (increase by 0.03 °C on average; from our unpublished data). Thus, we suggest that, with such improvements, this wind reduction method can still be effective and economical for use in field wind manipulating experiments.

Finally, we did not focus on the influence of the wind on the reproductive traits of plants, as the early harvest (needed for accurate measurement of *E*) did not allow for enough flowering or fruiting. However, it would be interesting to test whether the influence of wind could carry over to the next generation via the phenotypes of the seeds (e.g. through amount of reserves). Also, future experiments could test if the wind treatments could favor the survival and reproductive output of certain genotypes within a population of a species, thereby potentially also affecting the performance of future generations. Thus we suggest that our wind design could be the perfect candidate for further experiments to address this issue by collecting the seeds from plants from different wind treatments and then sow them to study subsequent generations.

## Conclusions

Our new experimental method to continuously increase wind speed, using wind-funneling baffles, enabled us to partially validate our null hypothesis that there are common whole-plant responses to wind stress across different plant species; strong wind significantly affected plant-size-related traits in our experiment. However, the response patterns of shoot traits, especially mechanical properties, to wind velocity were idiosyncratic among species. Furthermore, the wind effects on plant performance through changing leaf microclimate could be negative or positive depending on confounding factors including plant properties and local weather. In-depth experiments are needed to disentangle these effects of increasing wind on plant performance under different wind speeds achieved through different dimensions of wind-funneling baffles under field conditions.

## Supplementary Information

**Additional file 1: Table S1.** The specifications of all the instruments used in the experiment. **Fig. S1.** The distribution of wind direction of maximum wind speed from 2005 to 2015 at the meteorological station at Ordos ecological station. **Fig. S2.** Background environmental conditions during experimental period at Ordos ecological station. (A) Rainfall and temperature pattern within 2017. (B) Monthly mean and maximum wind velocity within the experimental period (April to October of 2017). (C) Daily mean and maximum wind velocity within the experimental period (April to October of 2017). (D) The distribution of wind direction of maximum wind speed from April to October of 2017. **Fig. S3.** Dynamics of air temperature (A), soil temperature (B), relative humidity (C) and volumetric water content (D) (mean ± SE) under different wind treatment over 24 h during the experiment period. D in the legend means decreased wind velocity treatment, CK means ambient wind velocity treatment, and I means increased wind velocity treatment. **Fig. S4.** Comparison of the wind treatment effects between the whole data set and the westerly-wind data subset extracted from the whole data set. D means decreased wind velocity treatment, CK means ambient wind velocity treatment, and I means increased wind velocity treatment. D-W, CK-W and I-W, respectively, are the westerly subsets of the data for the decreased, ambient and increased wind velocity treatments. **Fig. S5.** Response of various traits to different wind velocity treatments in four plant species: Leaf length (A), leaf width (B), leaf area (C), SLA (D), overall bifurcation ratio (E), Stem base diameter (F), and stem lignin content (G). Decrease means decreased wind velocity treatment, CK means ambient wind velocity treatment, and Increase means increased wind velocity treatment. Different lowercase letters indicate significant differences among the three treatments at P < 0.05. The error bars are plotted by means ± SE. **Fig. S6.** Results of the redundancy analysis (RDA) for the four plant species: distribution of each treatment of 4 plant species on the RDA1 × RDA2 plane. The relationship is significant (p < 0.01) based on 999 permutations. The adjusted *R*^2^ is 0.61. Suffix D of plant species name in the legend represents decrease wind velocity treatment, CK represented ambient wind velocity treatment, and I represented increase wind velocity treatment. The first dimension (RDA1), which describes 34.81% of the total variability, is positively correlated with stem cellulose content, root/shoot ratio and *I*, and negatively correlated with total biomass, shoot biomass, root biomass, plant height, leaf length, stem base diameter, leaf area, SLA, projected crown area, stem lignin content, *E*, *EI*, OBR and SBR_(1:2)._ The second dimension (RDA2), which explains 16.86% of the total variability, is positively correlated with *EI*, *I*, stem base diameter, leaf area, OBR and SBR_(1:2)_, while it is negatively correlated with root length, stem cellulose content, *E*, projected crown area and SLA. For trait abbreviations see the main text. **Fig. S7.** Application of wind manipulation design at community scale (4 m × 4 m quadrat, *A. ordosica* community) from our ongoing experiment. (A) Picture of the experiment set and detail of treatments. The Decreased wind velocity treatment (D) was implemented through a wind shield. The plastic chambers did not have a roof, so that ambient air could mix with that in the cubicles freely. Distances were kept between the wind shield and quadrat to avoid shading effect. There was no manipulation for the ambient treatment (CK). The Increased wind velocity treatment (I) was implemented through experimental wind baffles. We placed sheets at each side directed towards the North-East, North-West, South-East and South-West, respectively from each quadrat with plants, to converge and increase wind velocity. (B) Diurnal mean wind velocity pattern (mean ± SE) over 24 h under wind treatments. (C) Diurnal maximum wind velocity pattern (mean ± SE) over 24 h under wind treatments. (D) Effects of wind treatments. The black dashed line with the equation ($\text{y}=\frac{(s+\sqrt{2}l)}{s}x$) represents the ideal wind velocity that could be reached by acceleration. The black dashed line with the equation (*y* = *x*) represents the wind velocity in the treatment CK. Red dots and line represent the relationship between the temporally matched hourly maximum wind velocity in I and CK. Blue dots and line represent the relationship between the temporally matched hourly maximum wind velocity in D and CK. Regression equations and *R*^2^ are given.

## Data Availability

All data generated or analysed during this study are included in this published article [and its additional information files].
