# Supplementary material for: New field wind manipulation methodology reveals adaptive responses of steppe plants to increased and reduced wind speed
Source: Plant Methods. 2021 Jan 6;17:5. doi: 10.1186/s13007-020-00705-2 (PMC7788872; doi:10.1186/s13007-020-00705-2)
Supplement: Supplementary file 1 — Additional file 1: Table S1. The specifications of all the instruments used in the experiment. Fig. S1. The distribution of wind direction of maximum wind speed from 2005 to 2015 at the meteorological station at Ordos ecological station. Fig. S2. Background environmental conditions during experimental period at Ordos ecological station. (A) Rainfall and temperature pattern within 2017. (B) Monthly mean and maximum wind velocity within the experimental period (April to October of 2017). (C) Daily mean and maximum wind velocity within the experimental period (April to October of 2017). (D) The distribution of wind direction of maximum wind speed from April to October of 2017. Fig. S3. Dynamics of air temperature (A), soil temperature (B), relative humidity (C) and volumetric water content (D) (mean ± SE) under different wind treatment over 24 h during the experiment period. D in the legend means decreased wind velocity treatment, CK means ambient wind velocity treatment, and I means increased wind velocity treatment. Fig. S4. Comparison of the wind treatment effects between the whole data set and the westerly-wind data subset extracted from the whole data set. D means decreased wind velocity treatment, CK means ambient wind velocity treatment, and I means increased wind velocity treatment. D-W, CK-W and I-W, respectively, are the westerly subsets of the data for the decreased, ambient and increased wind velocity treatments. Fig. S5. Response of various traits to different wind velocity treatments in four plant species: Leaf length (A), leaf width (B), leaf area (C), SLA (D), overall bifurcation ratio (E), Stem base diameter (F), and stem lignin content (G). Decrease means decreased wind velocity treatment, CK means ambient wind velocity treatment, and Increase means increased wind velocity treatment. Different lowercase letters indicate significant differences among the three treatments at P < 0.05. The error bars are plotted by means ± SE. Fig. S6. Results of the red [file 13007_2020_705_MOESM1_ESM.pdf]

Table S1 The specifications of all the instruments used in the experiment.

Wind velocity, air temperature and relative humidity measurements	Manufacturer	Shenzhen frank electronics co. LTD., China
	Model type	AZ9671
	Wind Speed Range	0.6~32 m/s
	Wind Speed Accuracy	±(2% of reading+0.2 m/s)
	Air Temperature Range	-20~60 ℃
	Air Temperature Resolution	0.1 ℃
	Air Temperature Accuracy	±0.6 ℃ (-20~50 ℃), ±1.2 ℃ (at others)
	Air RH% Range	0.1%~99.9% RH
	Air RH% Resolution	0.1% RH
	Air RH% Accuracy	±3% RH (at 25 ℃ 10~90% RH, others ±5% RH)
	Temperature Response Time	60 seconds (typical)
	Air RH% Response Time	60 seconds (typical)
	Wet Bulb Temp. Display	-22~70 ℃
	Wet Bulb Temp. Resolution	0.1 ℃
	Operating Temperature	0~50 ℃
	Operating RH%	Humidity ＜80%
	Battery	AAA*4 PCS or 9V DC Adaptor
	Data Storage	2400 points
Soil volumetric water content and temperature measurements	Manufacturer	Decagon Devices, Inc, USA
	Model type	Em50 series data logger
	Sensor	Decagon 5TM VWC + Temp
	Soil Temperature Range	-40~60 ℃
	Soil Temperature Resolution:	0.1 ℃
	Soil Temperature Accuracy	±1 ℃
	Soil VWC Range	0~100%
	Soil VWC Resolution	0.0008 m^3/m^3 (0.08% VWC) from 0 to 50% VWC
	Soil VWC Accuracy	±0.01 to 0.02 m^3/m^3 (±1 to 2% VWC)
	Data Storage	36000 data scans

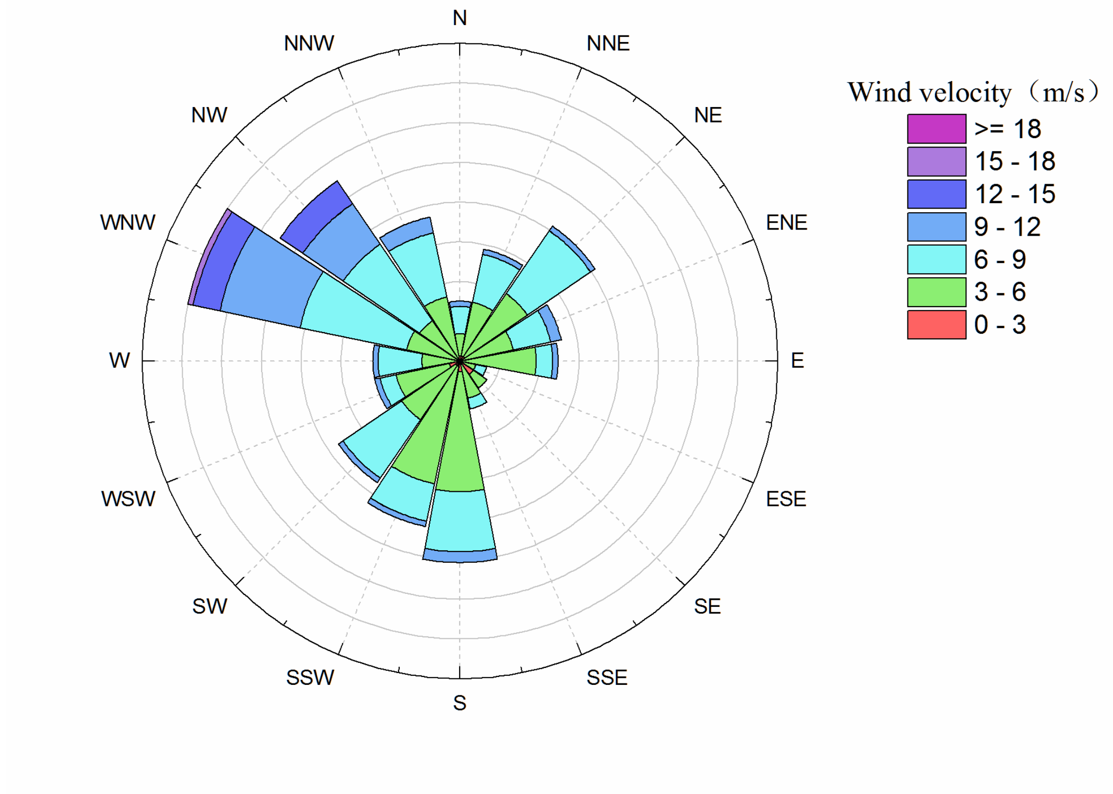

Fig. S1. The distribution of wind direction of maximum wind speed from 2005 to 2015 at the meteorological station at Ordos ecological station.

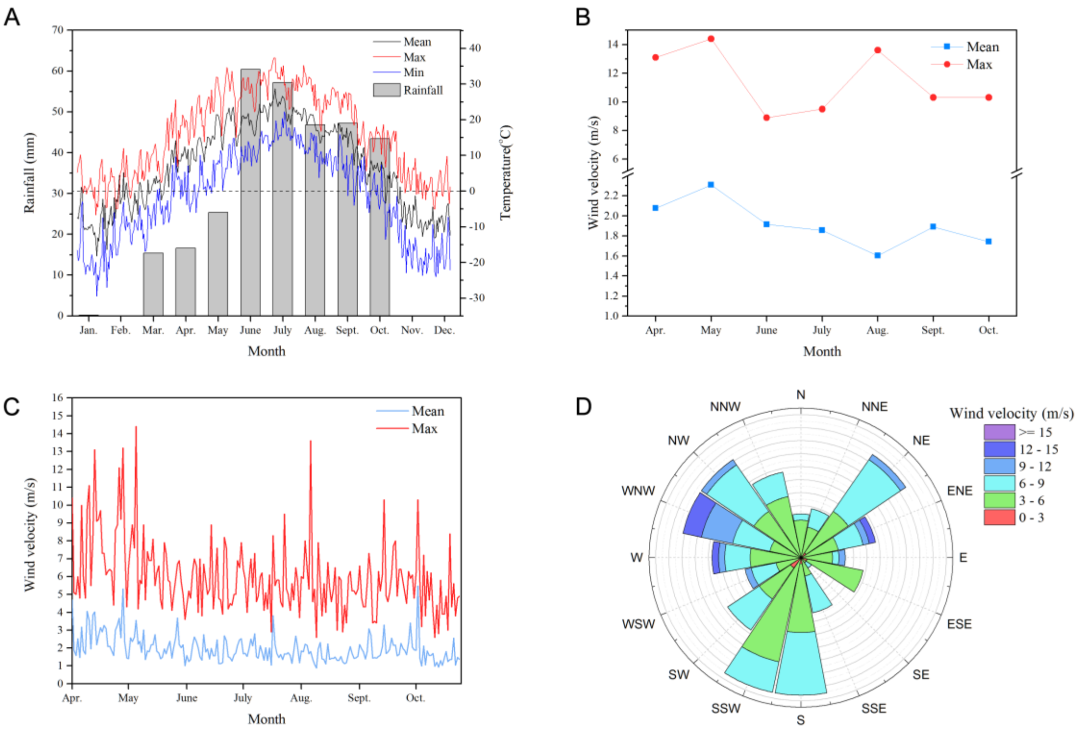

Fig. S2. Background environmental conditions during experimental period at Ordos ecological station. (A) Rainfall and temperature pattern within 2017. (B) Montly mean and maximum wind velocity within the experimental period (April to October of 2017). (C) Daily mean and maximum wind velocity within the experimental period (April to October of 2017). (D) The distribution of wind direction of maximum wind speed from April to October of 2017.

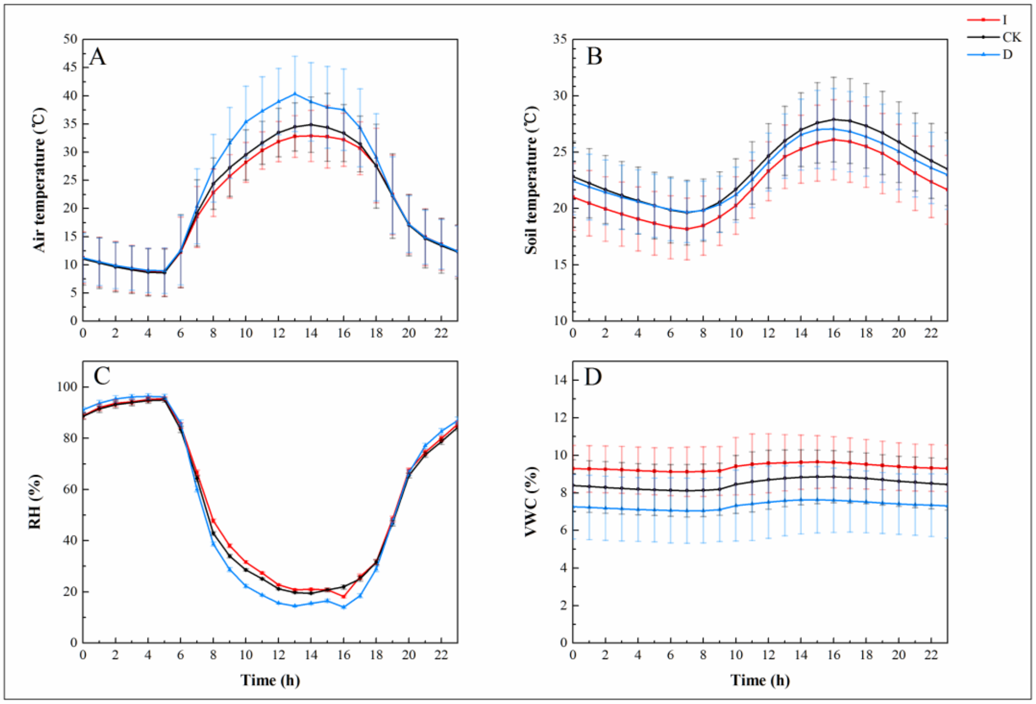

Fig. S3. Dynamics of air temperature (A), soil temperature (B), relative humidity (C) and volumetric water content (D) (mean ± SE) under different wind treatment over 24 hours during the experiment period. D in the legend means decreased wind velocity treatment, CK means ambient wind velocity treatment, and I means increased wind velocity treatment.

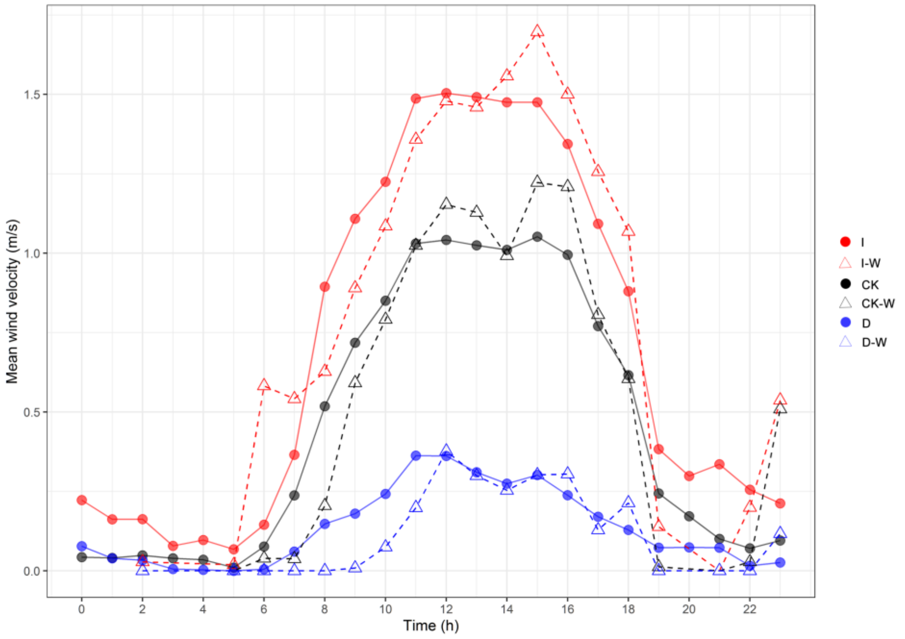

Fig. S4. Comparison of the wind treatment effects between the whole data set and the westerly-wind data subset extracted from the whole data set. D means decreased wind velocity treatment, CK means ambient wind velocity treatment, and I means increased wind velocity treatment. D-W, CK-W and I-W, respectively, are the westerly subsets of the data for the decreased, ambient and increased wind velocity treatments.

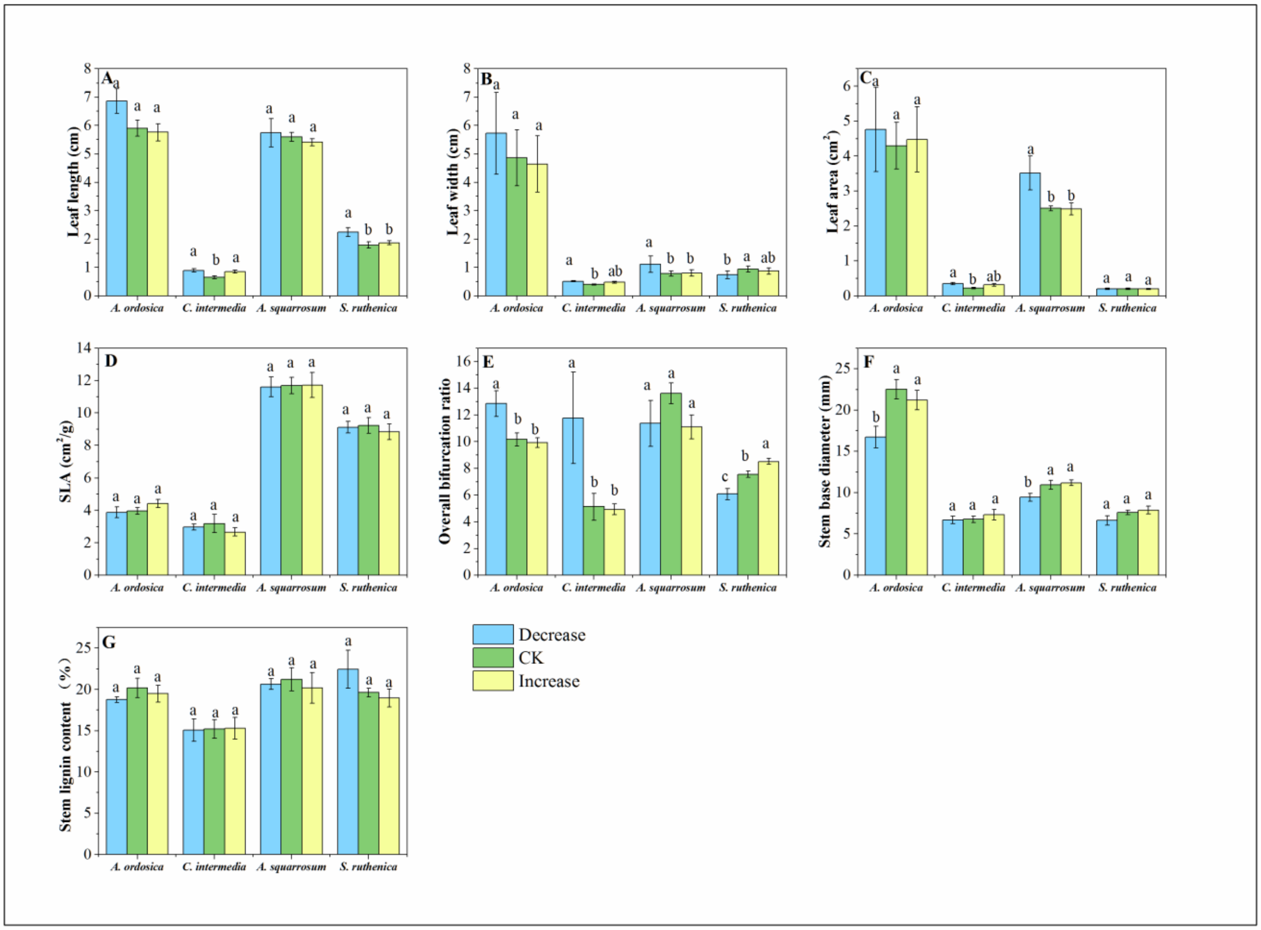

Fig. S5. Response of various traits to different wind velocity treatments in four plant species: Leaf length (A), leaf width (B), leaf area (C), SLA (D), overall bifurcation ratio (E), Stem base diameter (F), and stem lignin content (G). Decrease means decreased wind velocity treatment, CK means ambient wind velocity treatment, and Increase means increased wind velocity treatment. Different lowercase letters indicate significant differences among the three treatments at $P < 0.05$. The error bars are plotted by means ± SE.

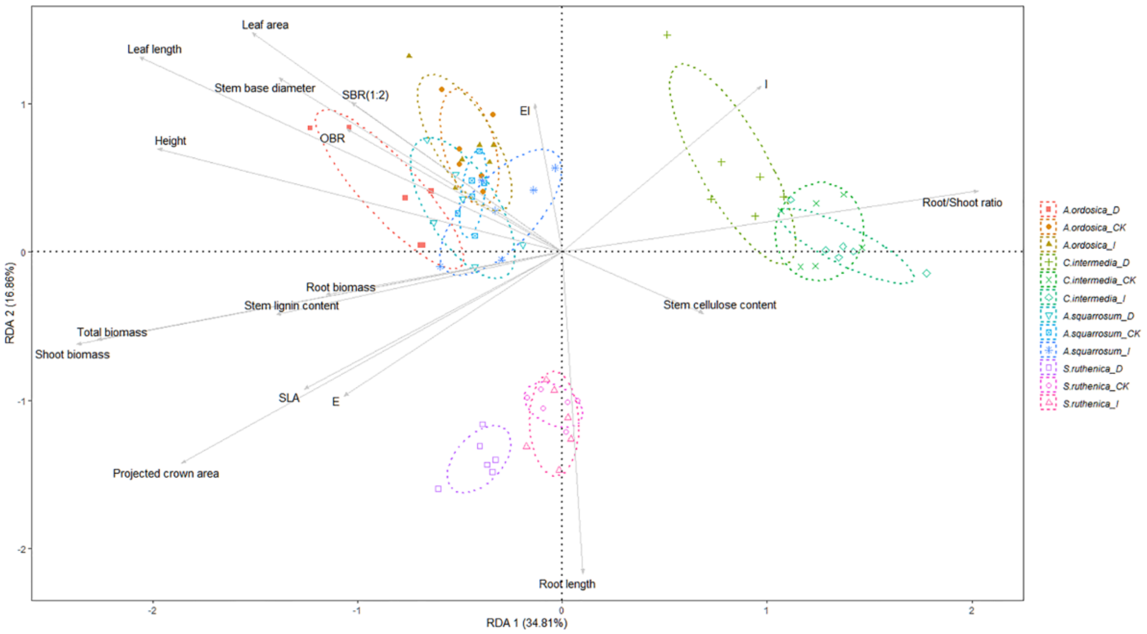

Fig. S6. Results of the redundancy analysis (RDA) for the four plant species: distribution of each treatment of 4 plant species on the RDA1×RDA2 plane. The relationship is significant ($P < 0.01$) based on 999 permutations. The adjusted R^2 is 0.61. Suffix D of plant species name in the legend represents decrease wind velocity treatment, CK represented ambient wind velocity treatment, and I represented increase wind velocity treatment. The first dimension (RDA1), which describes 34.81% of the total variability, is positively correlated with stem cellulose content, root/shoot ratio and *I*, and negatively correlated with total biomass, shoot biomass, root biomass, plant height, leaf length, stem base diameter, leaf area, SLA, projected crown area, stem lignin content, *E*, *EI*, OBR and $SBR_{(1:2)}$. The second dimension (RDA2), which explains 16.86% of the total variability, is positively correlated with *EI*, *I*, stem base diameter, leaf area, OBR and $SBR_{(1:2)}$, while it is negatively correlated with root length, stem cellulose content, *E*, projected crown area and SLA. For trait abbreviations see the main text.

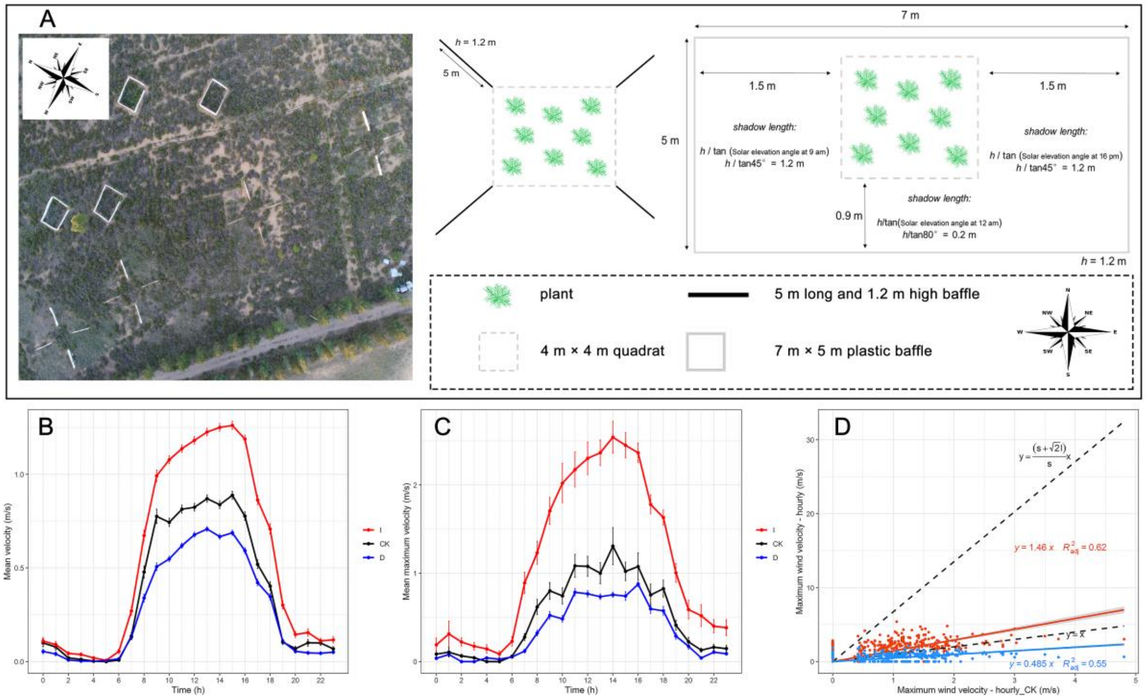

Fig. S7. Application of wind manipulation design at community scale (4 m × 4 m quadrat, *A.ordosica* community) from our ongoing experiment. (A) Picture of the experiment set and detail of treatments. The Decreased wind velocity treatment (D) was implemented through a wind shield. The plastic chambers did not have a roof, so that ambient air could mix with that in the cubicles freely. Distances were kept between the wind shield and quadrat to avoid shading effect. There was no manipulation for the ambient treatment (CK). The Increased wind velocity treatment (I) was implemented through experimental wind baffles. We placed sheets at each side directed towards the North-East, North-West, South-East and South-West, respectively from each quadrat with plants, to converge and increase wind velocity. (B) Diurnal mean wind velocity pattern (mean ± SE) over 24 h under wind treatments. (C) Diurnal maximum wind velocity pattern (mean ± SE) over 24 h under wind

treatments. (D) Effects of wind treatments. The black dashed line with the equation ($y = \frac{(s+\sqrt{2}l)}{s}x$) represents the ideal wind velocity that could be reached by acceleration. The black dashed line with the equation ($y = x$) represents the wind velocity in the treatment CK. Red dots and line represent the relationship between the temporally matched hourly maximum wind velocity in I and CK. Blue dots and line represent the relationship between the temporally matched hourly maximum wind velocity in D and CK. Regression equations and R^2 are given.
